# *Drosophila melanogaster* Selection for Survival of *Bacillus cereus* Infection: Life History Trait Indirect Responses

**DOI:** 10.1155/2012/935970

**Published:** 2012-10-09

**Authors:** Junjie Ma, Andrew K. Benson, Stephen D. Kachman, Zhen Hu, Lawrence G. Harshman

**Affiliations:** ^1^Department of Food Science and Technology, University of Nebraska Lincoln, Lincoln, NE 68583, USA; ^2^Department of Statistics, University of Nebraska Lincoln, Lincoln, NE 68583, USA; ^3^School of Biological Sciences, University of Nebraska Lincoln, Lincoln, NE 68588, USA

## Abstract

To study evolved resistance/tolerance in an insect model, we carried out an experimental evolution study using D. melanogaster and the opportunistic pathogen B. cereus as the agent of selection. The selected lines evolved a 3.0- to 3.3-log increase in the concentration of spores required for 50% mortality after 18–24 generations of selection. In the absence of any treatment, selected lines evolved an increase in egg production and delayed development time. The latter response could be interpreted as a cost of evolution. Alternatively, delayed development might have been a target of selection resulting in increased adult fat body function including production of antimicrobial peptides, and, incidentally, yolk production for oocytes and eggs. When treated with autoclaved spores, the egg production difference between selected and control lines was abolished, and this response was consistent with the hypothesis of a cost of an induced immune response. Treatment with autoclaved spores also reduced life span in some cases and elicited early-age mortality in the selected and wound-control lines both of which were consistent with the hypothesis of a cost associated with induction of immune responses. In general, assays on egg production yielded key outcomes including the negative effect of autoclaved spores on egg production.

## 1. Introduction

 Genetic selection in the laboratory provides a powerful tool for evolutionary analysis of complex traits [1]. It has been used to study many phenomena at different levels of biological organization including life histories, physiology, demography and population dynamics, behavior, form, sex, whole-genome evolution, altruism, and speciation [2]. Selection results in amplification of genetic differences between selected and control lines which is the basis of phenotypic differentiation. Often, correlated (indirect) responses to selection are of particular interest in these experiments as they can suggest tradeoffs between traits. For example, selection for increased *D. melanogaster* life span and late-age reproduction resulted in decreased early-age reproduction [3, 4]. The nature of tradeoffs between traits is an important topic in life history evolution [5].

 In the present study, the insect model *D. melanogaster* has been used in selection experiments for increased survival after bacterial infection. A previous study of responses in a laboratory selection experiment using *Pseudomonas aeruginosa* has examined the impact of *D. melanogaster* resistance on life history traits [6]. This study showed considerable costs in life span and larval survival as correlated responses to selection. While fly survival increased from 15% to 70% within ten generations in the selected lines, adult and larval viability decreased markedly relative to the control lines. In this selection experiment, microarray data indicated that a greater number of cellular immunity genes changed expression in the selected lines compared to the number of humoral immunity genes that changed, suggesting the relative importance of cellular immunity for resistance to *P. aeruginosa*. 

 A series of laboratory selection studies has also been conducted in which *Drosophila* evolved resistance against parasitoids [7, 8]. After 5 generations of selection the encapsulation frequency, important against parasitoids, increased from 5% to 60% in response to *A. tabida* and 0.5% to 45% in response to *L. boulardi*. Increased resistance to parasitoids was accompanied by a correlated evolutionary response in a number of traits, including doubling of the number of circulating haemocytes, smaller adult size, lower fecundity, reduced larval competitive ability, and increased pupal susceptibility [9, 10]. 

 One area of tremendous interest is the selection response of host organisms to infectious or zoonotic diseases. These diseases have significant impact on human and animal health, and understanding the evolutionary underpinnings of responses in humans may provide keys to alternative methods of prevention or intervention [11–16]. 

 To further understand the evolutionary implications of infectious disease resistance, and the potential for novel interventions, we have exploited *D. melanogaster* to study the effects of selection for resistance/tolerance to a spore-forming bacterial species (*Bacillus cereus*) which is closely related to the pathogenic spore-forming bioterrorism agent *Bacillus anthracis*. Because the spore is the most frequently encountered form of this organism (natural or otherwise), we used the spore form as a basis for selection. A strong response to selection for resistance to infection by spores was obtained, observed by a 3.3-log change in the number of spores required for approximately 50% mortality, within 24 generations. Here, we now demonstrate that life history traits were also affected as a consequence of selection and introduction of autoclaved spores. In wound-control and selected lines, exposure to autoclaved spores decreased life span. Selection was strongly positively correlated with egg production. When treated with autoclaved (dead) spores, the large difference between selected and control lines was abolished suggesting a cost of activating an immune response. There was a difference between untreated selected and control lines in progeny development time; the selected line progeny developed relatively slowly. After a series of matings were conducted to separate female and male effects, it was documented that exposure of selected and wound-control line males to autoclaved (dead) spores resulted in relatively rapid progeny development time. Finally, the selected and wound-control lines evolved heightened early-age mortality in response to the autoclaved spore treatment, which was interpreted as being consistent with the hypothesis of a cost associated with inducing an immune response. 

## 2. Materials and Methods

### 2.1. Fly Populations

 The procedure for establishing the base population as well as subpopulations used for selected and control lines in the present study was described in Schwasinger-Schmidt et al. [17]. Briefly, a large base population was maintained at approximately 10,000 individuals in an overlapping generation regime for approximately two years before being subdivided into 9 subpopulations (lines) for 5 generations in a similar population maintenance regime to that designed for the selected and control lines. Each rearing vial was seeded with 100 eggs to standardize density during the 5 discrete generations before the initiation of the selection experiment. In this artificial laboratory selection experiment, there were 9 lines which were separate outbred populations that evolved independently: selected lines, wound-control lines (punctured only with sterile H_2_O), and no-treatment lines. There were three replicates of each line type; replicates lines were independent populations that were subject to essentially the same conditions. The different sets of lines (selected, wound-control, and no perturbation) are referred to as “line types” in the present study. In *D. melanogaster* experimental evolution studies, “lines” are commonly used as a term to describe selected and control populations (e.g., see Rose 1984 and many subsequent *Drosophila* laboratory selection experiments).

### 2.2. Selection and Control Lines

**Figure 1:**
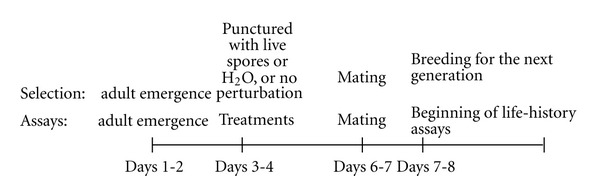
Timeline of the selection experiment and life-history assays. The life-history assays were designed to be conducted in parallel with the process used for the selected and control lines. Days were recorded as days posteclosion.

 Selection was conducted in a specific manner. *B. cereus* spores were used for selection. Spores were introduced into adults of the selected lines using a tungsten needle dipped into spores suspended in H_2_O. A concentration of spores that killed approximately 50% of the females and males was determined before the first generation of selection (2 × 10^6^ per mL). Every generation of selection the goal was to attain approximately 50% mortality after introduction of live spores. Each generation of selection, one thousand virgin females and the same number of virgin males were infected for each of three selected lines. After three days at room temperature, the number of survivors was determined for each selected line (S1, S2, and S3). Typically, there were 500 surviving females and 500 males per line. Surviving males and females within each line were counted and randomly placed in bottles to mate at a density of approximately 80 flies per bottles. Flies were kept in bottles for 24 hours to mate and lay eggs. The next day, eggs were collected from the bottles to initiate the following generation. Approximately, one hundred eggs were collected for each vial used to propagate the next generation. The vials with eggs were placed in 18°C for development. A temporal synopsis of the selected and control lines, and treatments, is presented in Figure 1. 

 There were two types of control lines. For the wound-control lines (CP for control punctured), females and males were punctured with sterile water without spores. Another set of control lines (CN) was used, and, in this case, there was no perturbation (no infection, no puncture). 

 The number of breeders used for each generation was the same for each set of matched lines of each type (selected, wound-control, and no perturbation). For example, the number of survivors after selection in selected line S2 was matched to the numbers of the control-punctured line CP2 and the no-perturbation line CN2. There were approximately 1000 flies (500 females and 500 males) used as breeders for each line each generation. 

### 2.3. Treatments

 All flies used from all lines were subjected to one of three treatments prior to life history assays. The treatments were similar, or the same, as used for selection and in the control lines (Figure 1). The autoclaved spore treatment (AS) was designed to induce an immune response with dead spores; it was analogous to administration of live spores. Live spores could not be used before assays at the level used for selection as they would cause excessive mortality. The treatment with sterile H_2_O was the same as used for the wound-control lines. Also, no treatment (NON) was the same condition as used for the no perturbation control lines.

### 2.4. Bacterial Culture and Spore Isolation

*Bacillus cereus* ATCC 10987 was used for spore purification using a step gradient of Renografin [18]. A single colony of *B. cereus* was inoculated in 25 mL of Difco Sporulation Medium (DSM) and incubated at 37°C on a rotary shaker (150 rpm) until mid-log phase. Inoculation of this culture into 2 L of DSM generated a 1 : 10 dilution which was followed by incubation at 37°C on the rotary shaker for 48 hours. The culture was centrifuged for 10 min at 10,000 ×g, and the supernatant gently discarded. The pellet was resuspended in 200 mL of 4°C sterile water followed by the same centrifugation procedure. The pellet was again resuspended in 200 mL of sterile water and stored overnight at 4°C. After repeating the centrifuge-resuspension-centrifuge procedure, greater than 90% of bright-field spores were observed under phase contrast microscopy. The pellet was resuspended in 20% Renografin and the suspension transferred to a 30 mL glass core tube with 15 mL of 50% Renografin. The spore suspension was centrifuged for 30 min at 4°C at 10,000 ×g. All layers containing vegetative cells were removed and the spore pellet retained. The pellet was resuspended in 10 mL of 4°C sterile water in an Oak Ridge tube. The spore suspension was centrifuged for 10 min at 10,000 ×g at 4°C. Trace amounts of Renografin were removed, by three washes with 4°C sterile water as described immediately above. The spore pellet was suspended in 2 mL of 4°C sterile water. The concentration of spores was determined by serial dilution and spread-plating.

 Spores were isolated twice during the portion of the selection experiment presented in this study. They were isolated the first time for selection generations 1–11. Also, they were isolated a second time for selection generations 12–24. Each preparation had very similar effects on mortality.

### 2.5. Life History Assays

 Conditions were standardized prior to life history assays. There was no selection for two generations prior to life history assays to minimize the impact of any effects that carry over from generation to generation such as maternal effects. There were nine lines and each was subject to three conditions prior to a life history assay (see Treatments section). Life span and egg production assays were conducted on flies two generations after relaxing selection at generation 18. Development time was conducted on flies derived from selection generation 24 after two generations of relaxed selection. 

 The age of flies assayed for life histories was designed to conform to the conditions of the selection experiment (Figure 1). The start of the life-history assays corresponded to the age of the flies used for the start of selection which was 7–9 days old (days posteclosion). Virgin flies (days 1-2) were collected from the breeding vials. At 3–5 days of adult life, the virgin flies were treated (punctured with autoclaved spores, punctured with water, and untreated). After three days, the level of survival was tabulated. Then, males and females were combined and allowed to mate and lay eggs for 24 hours before life-history assays were initiated. 

### 2.6. Life Span

 Life span, and all other life-history assays, were conducted with populations of flies held under standard conditions (25°C, 12 : 12 L : D). The cages used for the life span assay were made out of quart-size plastic containers. The lid had mesh inserted for ventilation. There was a grommet in the side of each container with a tube allowing for replacement of used food vials with fresh food vials every three days. A rubber patch was sewn into the opposite side of each container, and it had a slash in it to allow insertion of a Pasteur pipette. The pipette was used to aspirate dead flies from the bottom of the cage allowing them to be removed and recorded every three days. Each cage initially received 30 flies of the same sex that had been allowed to mate for 24 hours prior to the assay. There were four replicate cages for each sex and treatment for all lines. The cages were monitored until all flies were dead.

### 2.7. Egg and Progeny Production

 Egg production was recorded for all lines and treatment combinations. Twenty mated females (males were discarded after the 24-hour mating period) from each line and treatment were placed individually in vials at 25°C. Females were transferred to new vials each day for 49 days at which time almost all of the eggs were produced.

 For determination of adult progeny numbers, after eggs were counted, the replicate vials for all lines and treatments were placed at 18°C. Emergent progeny was counted from all vials until all adults emerged.

### 2.8. Progeny Development Time

 The design for development time was more complicated than for life span or fecundity as additional combinations of matings were used. For each line, five different *F*_1_ crosses were employed to parse out male and female treatment effects on progeny development time (Table 1). For example, females treated with autoclaved spores were mated to untreated males which allows for assessment of the effect of female treatment on progeny development time. The reciprocal mating allows for assessment of the effect of the autoclaved spore treatment on males with progeny development time as the outcome. These were the first two matings. Similarly, the effect of puncturing females or males with sterile H_2_O was evaluated by reciprocal crosses with progeny development time as the outcome. These were the third and fourth matings. The fifth mating allowed for assessment of progeny development time when neither male nor female was treated. There were six replicates of each of the five different *F*_1_ matings. The five matings were used for all lines to investigate progeny development time. Approximately 100 eggs were collected from each cross after the 24-hour mating period and placed in each vial to control larval density. All of the vials (matings, lines) were randomized with respect to the order that matings were initiated. The time of first emergence of adults was *t*_0_ for the progeny development time assay. The counting period of emerged progeny continued well beyond the time when no additional adults eclosed.

### 2.9. Survival after Administration of Autoclaved Spores

 There was a three-day period after treatments, when survival was monitored before the beginning of life-history assays. In Figure 1, this period is shown as occurring after the administration of treatments and before mating (posteclosion ages 3-4 to 6-7). Mortality during this period was considered separately from life span or other life-history assays. This data was tabulated and statistically analyzed.

### 2.10. Statistical Analysis

 The data was analyzed by ANOVA using SAS version 9.3 (SAS, 2009). The data was treated as continuous. A mixed model analysis of variance was used with line types and treatments as fixed effects. Random effects were derived from variation among the three replicate lines of the same type. Variation among lines of the same type was nested within fixed effects for the analysis. For each sex, all lines and treatments were analyzed with one ANOVA for every life history trait. Any significant, or nonsignificant, interaction terms were derived from an analysis using the full model. Residuals were examined by QQ-plots and histograms in order to detect deviations from normality. 

 For life span, the average survival time, median survival time, first quartile survival (25% mortality), and time to third quartile survival (75% mortality) of females or males were used for statistical analysis. For development time, the average progeny eclosion time was used for statistical analysis. Total egg and progeny numbers were used for statistical analysis of reproduction. Any statistically significant interactions between treatments and lines were explicitly described in the present study in terms of the pattern of the data.

## 3. Results

### 3.1. Statistical Analyses

 The statistical analysis was conducted using mixed model ANOVAs. The degrees of freedom and  *F*  values are presented in supplemental tables (see S1a—life span, S1b—egg and progeny production, and S1c—progeny developmental time in Supplementary material available online at doi:10.1155/2012/935970). Examination of QQ-plots and histograms for all of the data indicated no major deviations from normality. 

### 3.2. Direct Response to Selection

**Figure 2:**
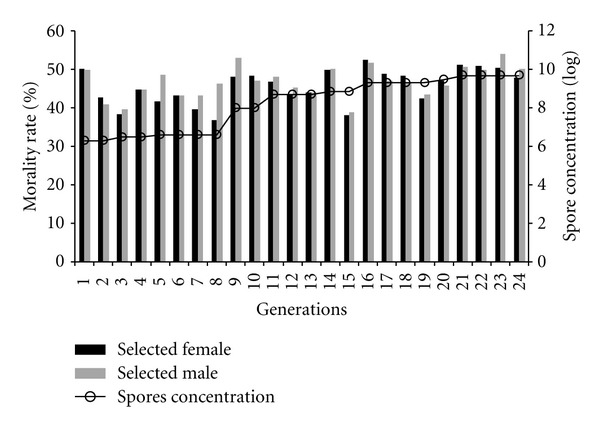
Direct response to selection for survival after *B. cereus* live spore infection. After 24 generations of approximately 50% mortality of females and males, the concentration of spores required for this level of mortality increased by 3.3log.

 In order to exert steady selective pressure across multiple generations, the spore concentration used for selection at each generation was increased to attain 50% mortality. As shown in Figure 2, this approach led to a steady incremental response to selection over 24 generations, producing a 3.3-log increase in spore concentration necessary for 50% mortality. The response to selection was almost log-linear. In some cases, there was an increase in survival in the next generation after relatively strong selection in the previous generations (generations 8 to 9 and generations 15 to 16). This might have resulted from selecting to greater degree in generations 8 and 15, hypothetically resulting in a genetically more resistance subset of the population, which could have responded in the next generation by elevated survival in generations 9 and 16, respectively. 

 For the S1 selected line, a late generation of selection (generation 36) was tested for resistance by introducing the spore concentration (2 × 10^6^ per mL) used for the first generation of selection into 100 females and 100 males. There was no mortality after the standard three-day observation period. This result complements the observation of an incremental response over 24 generations and provides further evidence of a direct response to selection. 

### 3.3. Indirect Responses to Selection

 The *P* values for all of the line types (selected, wound-control, and no perturbation) and treatments (autoclaved spores introduction by puncturing, punctured with H_2_O, and no treatment) are presented in Table 1. 

 For reporting results in the following text, there typically are separate subheadings for untreated samples and samples treated with autoclaved spores. However, the sterile H_2_O treatment was sometimes also included. Untreated lines represent the consequences of evolution, whereas lines treated with autoclaved spores hypothetically represent the consequences of eliciting an induced immune response. In the parlance of McKean and Lazaro [19], untreated lines are analogous to “standing defense,” and autoclaved spore-treated lines were designed to be analogous to “deployment.”

### 3.4. Life Span

#### 3.4.1. Untreated Lines

 The average percent survival (life span) of untreated selected and control-line females was determined. Overall, there were no line effects for females or for males. The survival curves for both sexes when untreated are presented in supplemental figures (S2a and b).

#### 3.4.2. Autoclaved Spore—Treated Lines

**Figure 3:**
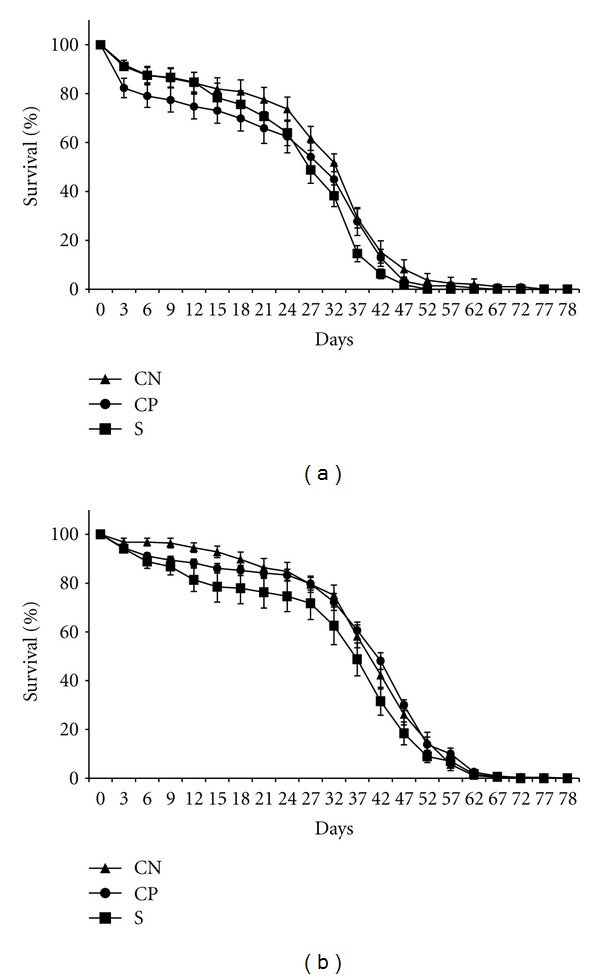
(a) Average percentage survival of adult females from the selected and control lines treated with autoclaved spores. The mean was determined from the replicate lines of the same type: S—selected lines, CP—lines punctured with H_2_O (wound control), and CN—no perturbation lines. (b) Average percentage survival of adult males from the selected and control lines treated with autoclaved spores. The mean was determined from the replicate lines of the same type: S—selected lines, CP—lines punctured with H_2_O (wound control), and CN—no perturbation lines.

 The average percent survival (life span) of the autoclaved spore treatment applied to selected and control females is presented in Figure 3(a). The average percent survival (life span) of the autoclaved spore treatment applied to selected and control males is presented in Figure 3(b). The average survival time after treatments is presented in Table 2(a) (females) and Table 2(b) (males). Overall, there was a statistically significant effect of treatments on females (*P* < 0.0001) and males (*P* = 0.0003). Treatment with autoclaved spores reduced mean female life span in the selected lines (27.65 days) and wound-control lines (27.92 days) compared to the no-perturbation lines (31.35 days). The decrease in selected female life span was approximately 10%. Autoclaved spores reduced mean male survival in the selected lines (35.17 days), compared to wound-control (40.10 days) and no-perturbation (40.50) lines. Autoclaved spores decreased the survival of selected males by 14.5%. In neither females nor males was there a statistically significant interaction between treatments and line types. In general, the administration of autoclaved spores reduced life span in the wound-control lines and for selected line males. This observation is consistent with the hypothesis of the cost of an induced immune response (deployment). 

### 3.5. Egg and Progeny Production

#### 3.5.1. Untreated Lines—Egg Production

**Figure 4:**
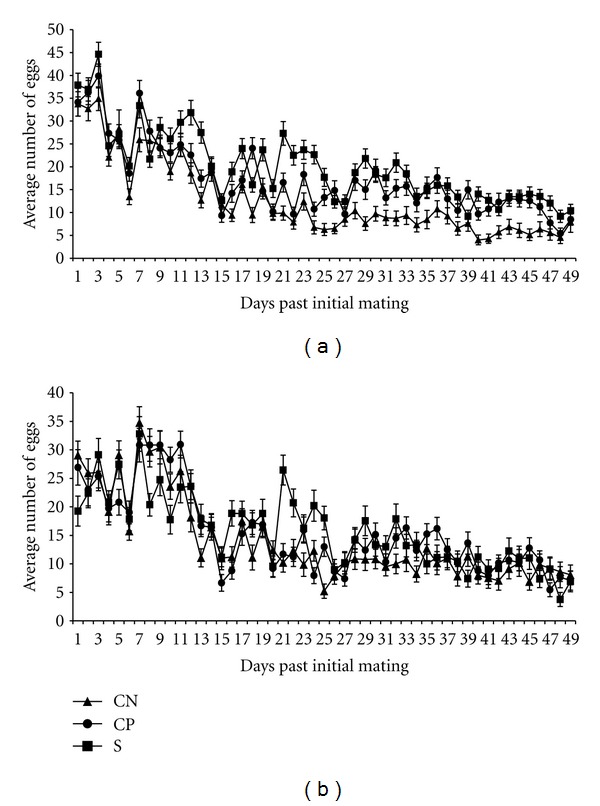
(a) Average daily egg production among line types when there was no treatment of adult females and males. The mean was determined from the replicate lines of the same type: S—selected lines, CP—lines punctured with H_2_O, and CN—no perturbation lines. (b) Average daily egg production among lines when adult females and males from the same line were treated with autoclaved spores. The mean was determined from the replicate lines of the same type: S—selected lines, CP—lines punctured with H_2_O, and CN—no perturbation lines.

 Total average egg production number is shown in Table 3 for lines and per female per day in Figure 4(a). Overall, for total egg production, there were statistically significant line (*P* = 0.0215), treatment (*P* = 0.0039), and line by treatment interaction (*P* = 0.0280) effects (Table 2). A major difference between line types was observed when there was no treatment (*P* < 0.0001). Selected lines produced a markedly high number of eggs (874), wound-control lines were intermediate (737), and no-perturbation lines produced the lowest number eggs (561) (Table 3). The selected line produced 19% more eggs than the wound-control lines and 56% more eggs than the no-perturbation lines. The wound-control lines produced 31% more eggs than the no-perturbation lines. 

#### 3.5.2. Autoclaved Spore Treatment—Egg Production

 Egg production for autoclaved spore-treated females (and males) for all line types is presented in Table 3 and Figure 4(b). The effect of treatment with autoclaved spores was to markedly reduce average egg production in the selected lines (588 eggs) and in the wound-control lines (636 eggs) (Table 3). The effect of treatment was statistically significant for the selected lines (*P* = 0.0001), but not the wound-control lines. There was no reduction of average total egg production in the no-perturbation control lines as a result of treatment with autoclaved spores (Table 3). The statistically significant interaction resulted from the decrease in egg production in the selected and wound-control lines, but not the no-perturbation lines. This reduction in total egg production after treatment with autoclaved spores, especially acute in the selected lines, is consistent with the hypothesis of a cost associated with induction of an immune response (deployment). 

#### 3.5.3. Untreated Lines—Progeny Production

Table 3 presents the progeny number for lines and treatments. There were only marginal statistically significant differences in progeny production among line types (*P* = 0.0961, Table 2). In dramatic contrast to egg production, the selected lines did not produce significantly more progeny (Table 2). Progeny production is dependent on the number of sperm transferred to females and stored after the 24-hour mating period, and this did not differ appreciably among line types. 

#### 3.5.4. Autoclaved Spore and Sterile H_2_O Treatments—Progeny Production

Table 3 presents the progeny numbers after treatment with autoclaved spores or sterile H_2_O. The treatment effects were statistically significant overall (*P* = 0.0005, Table 2). Autoclaved spore treatment resulted in the fewest number of progeny (CN and S) and punctured with sterile H_2_O resulted in the greatest number (CN and CP) (Table 3). There were no statistically significant interactions between line types and treatments. 

### 3.6. Progeny Development Time

 Progeny development time represents egg to adult emergence time. As described in the materials and methods and Table 4, four different crosses allowed us to separate the progeny development time effects of treatment (autoclaved spores or sterile H_2_O) on progenitor females or males. The fifth mating allowed us to evaluate an absence of treatments (mating pattern E in Table 4). All adults from all lines were subjected to each of the five crosses (A–E in Table 4). 

#### 3.6.1. Untreated Adult Females and Males

**Figure 5:**
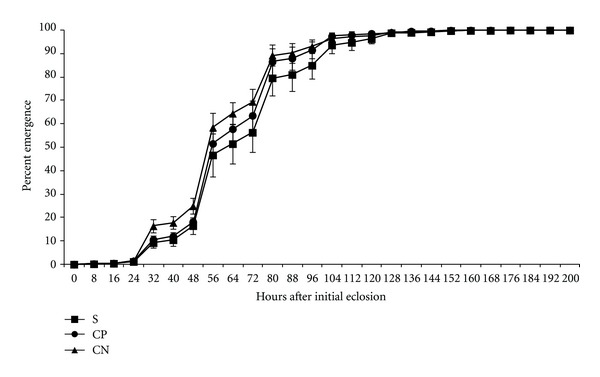
Average adult progeny emergence (eclosion) for line types when females and males were untreated. Line types: S—selected lines, CP—lines punctured with H_2_O each generation, and CN—no perturbation lines.

Figure 5 presents the cumulative percentage progeny emergence per time period for all lines types when there was no adult fly treatment. The progeny development time for selected line flies was slowest (Table 4). Statistically significant effects on progeny development were observed when there was no adult treatment (*P* = 0.0098, Table 2). This observation is consistent with the hypothesis of an evolved cost (standing defense) which is manifest as delayed progeny development time. However, an alternate hypothesis is presented in the discussion.

#### 3.6.2. Treatment of Adult Females and Males

**Figure 6:**
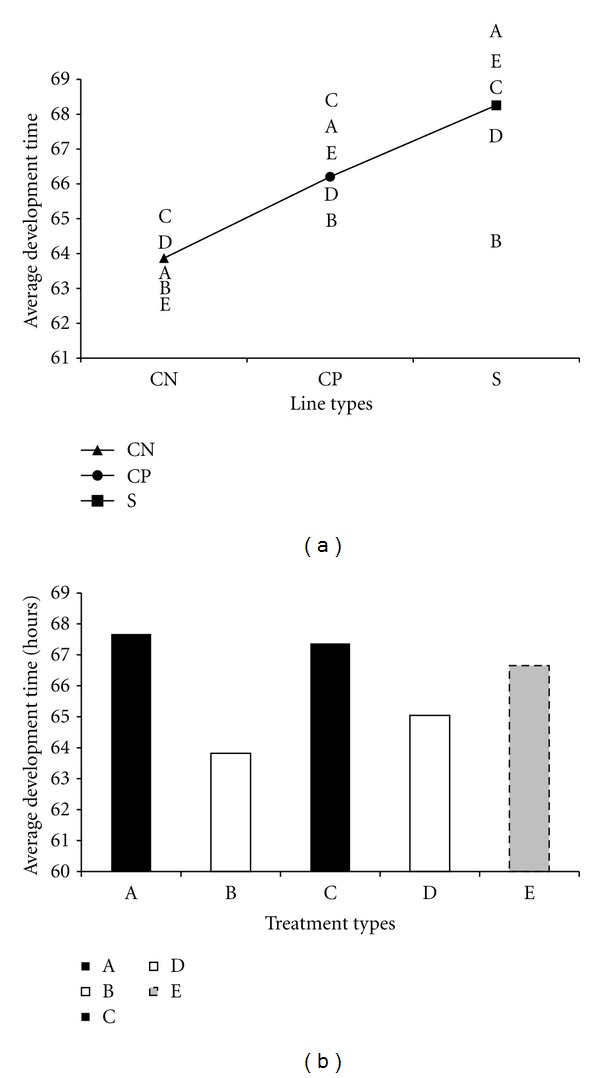
(a) Line graph of progeny development time from different *F*_1_ matings shown for each line type. Line types: S—selected lines, CP—lines punctured with H_2_O each generation, and CN—no perturbation lines. The *F*_1_ treatments are described in Table 1 and reiterated here: A—only females treated with autoclaved spores, B—only males treated with autoclaved spores, C—only females treated with sterile H_2_O, D—only males treated with sterile H_2_O, and E—both sexes untreated. (b) Bar graph of overall means of progeny development time for each *F*_1_ mating. The *F*_1_ matings are described in Table 1 and reiterated here: A—only females treated with autoclaved spores, B—only males treated with autoclaved spores, C—only females treated with sterile H_2_O, D—only males treated with sterile H_2_O, and E—both sexes untreated.

 There was a statistically significant sex-dependent effect of adult treatment on progeny development time (*P* = 0.0051, Table 2). When females were treated with autoclaved spores, or sterile H_2_O, progeny development time was similar to that observed when neither sex was treated (Figures 6(a) and 6(b)). The rank order of progeny development time was selected lines slowest, wound-control lines intermediate, and no-perturbation lines fastest. The grand mean value for male treatments (autoclaved spores and sterile H_2_O, treatments B and D in Table 4) was 64.4. This value was similar to 66.7 which was the mean for untreated females and males (mating E in Table 4). Thus, when males were treated, the adult progeny emergence time dropped below the level of the untreated lines (Figures 6(a) and 6(b)). This male effect was more pronounced in the selected lines (*P* = 0.0012), again emphasizing the impact of evolution for resistance in these lines. 

 The number of adult progeny emerging was tabulated for each population and treatment to evaluate if there was a density effect on progeny development time. Comparing the number of progeny from treated males (treatments B and D) with treated females (treatments A and C), there was a 0.9% difference. The effect of treated males on progeny development time was not due to larval density as inferred from the number of adult progeny that eclosed. 

### 3.7. Three-Day Survival after Administration of Autoclaved Spores

**Figure 7:**
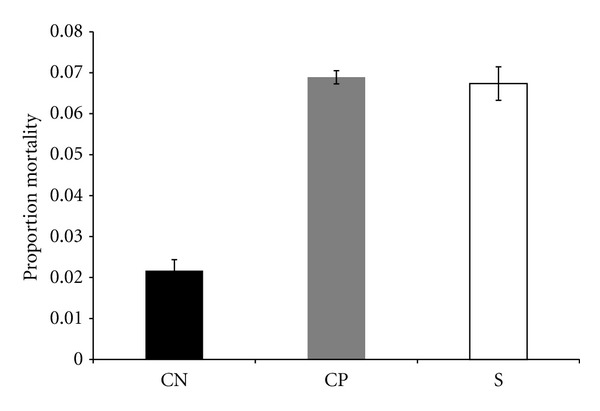
Average proportion mortality after treatment with autoclaved spores. The average was obtained by pooling all of the variates for all lines of the same type. S—selected lines, CP—lines punctured with H_2_O (wound control), CN—no perturbation lines.

 The selected lines and wound-control lines evolved an increase in mortality in the three-day period after autoclaved spores were introduced (Figure 7). Importantly, this three-day period occurred before the start of life-history assays (Figure 1). The no-perturbation control lines exhibited 2.11% mortality after introduction of the autoclaved spores, whereas the selected lines (6.74% mortality) and wound-control lines (6.89% mortality) showed a marked increase in relative mortality after introduction of autoclaved spores. This difference was statistically significant (*P* = 0.0003). The increase in mortality of the selected lines compared to no-perturbation lines was 3.19-fold and 3.27-fold for the selected and wound-control lines, respectively. It is important to emphasize that there was almost no mortality after the flies were only wounded (sterile H_2_O treatment). Thus, the effect shown in Figure 7 was not an evolved response to mortality from wounding. In general, there was an early-age spike of mortality in the selected lines and wound-control lines after autoclaved spore administration. This observation is consistent with the hypothesis that there was cost of inducing an immune response (deployment). 

## 4. Discussion

Our work here has established that *D. melanogaster* can evolve high levels of resistance to *B. cereus* spores. Specifically, a 3.3-log change in the number of spores required for 50% mortality over 24 generations was documented. After a substantial direct response to selection, we investigated life history trait indirect responses to selection which was the focus of this study. Extrapolating from McKean and Lazzaro [19], selection responses *per se* represent the “standing defense” which can exert a cost. Moreover, when an inducer of the immune response was introduced, autoclaved spores in the present study, then “deployment” is the cost of maintaining the immune system in an activated state under conditions where its function is unnecessary and indeed detrimental, and this can also exert a cost [19]. This is in many ways analogous to the detrimental effects of inflammation on multiple systems in humans (e.g., metabolic disorders, IBD, and arthritis) as illustrated by the inflammatory bowel diseases [20]. In the present selection experiment, we could compare the cost of selection under noninducing conditions and differentiate this cost from that observed when a putative inducer (autoclaved spores) was introduced into flies. 

 There were six principle indirect responses in the present study. The first was that life span was reduced after introduction of autoclaved spores into males in the selected lines, and females and males in the wound-control lines. This observation is consistent with the hypothesis of a cost associated with induction of an elevated immune response. Second, egg production was markedly elevated in untreated selected lines and to a lesser degree in untreated wound-control lines. There might have been increased titers of one or more hormones that could have a mutually stimulatory effect on reproduction and the immune response. Or, selection for increased adult fat body could underlie this indirect response as described below in the paragraph starting with “An alternative perspective….” Third, the relatively high egg production in the untreated selected lines, and to lesser degree in the wound-control lines, was abolished after introduction of autoclaved spores. The observation is consistent with the hypothesis of a cost of induced immunity in these lines. Fourth, untreated selected lines exhibited relatively slow development time. This observation is consistent with the hypothesis of an evolved cost associated with a high degree of resistance to live *B. cereus* spores (standing defense). However, there is an alternate adaptive hypothesis that could explain delayed development (see the paragraph below “An alternative perspective…”). Fifth, the progeny of selected line males exposed to autoclaved spores, then mated to untreated females, developed relatively rapidly. The biological interpretation of this observation is not obvious, but it might reveal a novel effect of male accessory gland secretions. Sixth, a pathological outcome (decreased early-age survival) was observed in the selected and wound-control lines after treatment with autoclaved spores. This observation is consistent with the hypothesis of a deleterious effect associated with induction of the immune system (deployment). This was an intriguing evolutionary observation as it suggests how the selection response for elevated immunity could eventually be constrained. Overall, there were multiple instances in which deployment was associated with a cost, and one case in which standing defense was potentially associated with a cost.

Evolution of resistance to bacterial spores has been observed in *Aedes aegypti* populations when *Bacillus thuringiensis* subspecies *israelensis* was the pathogen [21]. In this instance, resistance likely evolved due to changes in toxin-gut receptor interactions as observed in the diamond back moth [22]. In the present study, the level of resistance to *B. cereus* spores was many-fold greater than previously observed for selection on *A. aegypti* using *B. thuringiensis* spores. The direct response to selection in the present study was unprecedented for insect resistance to *Bacillus spores*.

The absence of a negative effect on life span in the selected lines in the present study differs from the selection experiment on *D. melanogaster* for resistance to *P. aeruginosa* [6], in which life span was negatively affected. There were appreciable differences between the selection process in Ye et al. [6] and the present study that could account for the different outcomes. This includes the use of vegetative cells versus spores, use of a gram-negative versus gram-positive pathogen, the size of the selected and control populations, and using approximately 50% mortality level for every generation of selection in the present study. Another possible explanation might be that *P. aeruginosa* is a more virulent pathogen and could exert a greater effect on life span than did *B. cereus* spores in the present study.

Generally, a negative relationship between reproduction and immunity has been observed [19, 23]. For example, decreased reproductive activity is associated with increased immunity in the cricket and in *Drosophila* [24, 25]. Thus, the major increase in egg production in the untreated selected lines in the present study was an unexpected outcome. The endocrine system of *D. melanogaster *might provide insight into understanding this phenomenon. In females, the evolution of elevated juvenile hormone (JH) could stimulate egg production, but elevated JH would also be expected to suppress immunity [26]. It is possible that male accessory gland proteins and peptides could have evolved to stimulate female egg production as there are a number of peptides in the male ejaculate that have this effect [27]. Almost all of the male ejaculate effect on female egg production is a result of the action of the sex-peptide [28]. However, the sex-peptide also stimulates JH production [28], and this would tend to suppress immunity. Importantly, the elevated egg production response in the selected lines was manifest for most of the reproductive life time. Thus, it does not seem likely that elevated egg production was a male effect resulting from mating for 24 hours relatively early in life. Another endocrine candidate is insulin, insulin-like signaling (ISS). ISS mutations in *Drosophila* result in sterility or extremely low levels of oocyte production [29, 30]. ISS in *D. melanogaster* is known to be the key hormone in the endocrine control of vitellogenic oocyte development [31], and ISS mediates the signals from nutrients to upregulate egg production [32]. However, the effect of elevated insulin signaling on *D. melanogaster* is to suppress innate immunity in this species [33]. At present, it is not clear which hormone in adult *D. melanogaster* could have caused a positive correlation between evolved elevated survival after *B. cereus* infection and high levels of egg production in the untreated selected lines. 

 An alternative perspective, perhaps involving hormones, might provide an explanation for the strong positive correlation between the evolution of a high degree of resistance to spores and elevated egg production. Specifically, part of the evolved response to spore infection might have been a delayed development rate which was observed in the selected lines in the present study (Figure 5, Table 4). If this delay resulted in an increase in adult fat body tissue, then there would be more of the tissue that principally secretes antimicrobial peptides. It is known that an acceleration of development time results in lower levels of fat in *D. melanogaster* larvae and adults [34, 35], and, conversely, a delay in development is expected to increase adult fat content. Hormones could mediate delayed development if juvenile hormone titers were relatively high in larval and pupal stages, and relatively low in the adult stage to avoid suppression of the immune response in the life stage at which selection occurred. The hypothesis of delayed development time as an adaptation could explain the positive correlation between egg production and the response to selection. Moreover, the interpretation of the delay in development as a standing defense cost of selection would be replaced by delay of development as an adaptive response.

Introduction of autoclaved spores abolished the relatively high egg production in the selected and wound-control lines; after the autoclaved spore treatment, these lines did not produce more eggs than the no-perturbation lines. This result is consistent with the hypothesis of a cost of inducing (deployment) of the immune response. 

There was a striking difference between the results for egg production in the present study versus the results for progeny production. Progeny production is a much different trait. For example, the total number of progeny is limited by the number of sperm stored after a mating. Lifetime egg production does not have this kind of constraint and the numbers can be much greater. 

Two examples of changes in progeny development time were observed in the present study. The selected lines exhibited delayed development when the *F*_1_ generation was untreated. This observation is consistent with the hypothesis of an evolved (standing) cost manifest in progeny development. In the context of a species that develops rapidly, delayed development could be interpreted as a cost. However, delayed development time could be an adaptation as described in a paragraph above (“An alternative perspective…”). The implications of observing accelerated development after treatment of adult males with autoclaved spores or sterile H_2_O (Figures 6(a) and 6(b)) are less clear. It might be the case that the effect of male seminal fluids is normally to delay the development time of progeny. In this scenario, when males are impacted by wounding or spores, then the normal male effect might be blocked. However, an effect of male seminal fluids on progeny development time is not established; the present study may suggest a novel function for male seminal fluids.

An interesting evolutionary observation in the present study was that introduction of autoclaved spores into the selected and wound-control lines resulted in elevated early-age mortality. This effect was observed during the three-day period when mortality was monitored after introduction of autoclaved spores, puncture with H_2_O, or no treatment before life-history assays were conducted (Figure 1). In two line types, selected and wound-control, exposure to dead spores resulted in relatively high mortality. One hypothesis is that the wound-control lines evolved a similar response to the selected lines because wounding each generation activated immune responses in these lines. If these responses were costly each generation, then the wound-control lines might have evolved indirect responses that were similar to the selected lines. Another hypothesis is that the short-term mortality response of the selected lines was entirely due to wounding. However, there was no increase in mortality in either line type after treatment with sterile H_2_O which is the wounding alone treatment. In general, the evolved higher mortality in selected and wound-control lines in response to autoclaved spores suggests one way in which selection for immunity can be constrained which is through counter-tending negative effects that oppose the direct response to selection.

 In this study, the effect of exposure to autoclaved spores is consistent with the hypothesis of a cost of induced immunity (deployment) on life history traits. This was observed for life span, egg production, and early-age mortality. Overall, the observation of an inducible cost resulting from the introduction of autoclaved spores is consistent with the hypothesis that the selected and wound-control lines have evolved to become hyper-inducible in response to autoclaved spores. The evolution of inducible responses apparently is a general response to selection in experimental evolution studies [36, 37] and perhaps in natural populations. 

 An extension of the studies described here will provide insight into the evolution of *B. cereus* spore infection resistance and/or tolerance in *D. melanogaster*. Whole-genome mapping of the responses to selection is underway as is a whole-genome transcriptome study. Through these approaches, and by other means to investigate resistance/tolerance, there is potential to increase our understanding of mechanisms underlying the dramatic response of *D. melanogaster* to selection by *Bacillus cereus* spores. Novel mechanisms of resistance/tolerance may emerge from these studies.

## Supplementary Material

In the supplementary material, S 1a, S1b and S1c tables present the F values and degrees of freedom from the statistical analysis with using a mixed model ANOVA of female and male survival time (a), egg and progeny production (b) and progeny development time (c). Figures S2a and S2b present average percentage survival of the selected and control lines in which adult females (a) and males (b) were untreated. .

## Figures and Tables

**Table 1:** Mating design for the progeny development time assay.

	Mating for progeny development time
One sex treated with autoclaved spores	A treated females × untreated males
	B treated males × untreated females
	
One sex types treated with sterile H_2_O	C treated females × untreated males
	D treated males × untreated females
	
No treatment	E untreated females × untreated males

All line types (selected—S, puncture control—CP, and no perturbation—CN) were used for crosses A, B, C, D, and E. The average emergence (eclosion) time of progeny was determined for each cross (A–E).

**Table 2:** *P* values from comparisons (average survival of females, average survival of males, total egg production, total progeny production, and average progeny development time) of treatments within lines of the same type and comparisons of line types for each treatment.

Overall effects	Lines/treatments	Comparisons	*P* value of female life span	*P* value of male life span	*P* value of egg production	*P* value of progeny production	*P* value of progeny development time
Lines			0.4444	0.5330	0.0215	0.0961	0.1457
Treatments			<0.0001	0.0003	0.0039	0.0005	0.0051
Lines* treatments			0.1027	0.1603	0.0280	0.1535	0.1790
	Lines						
	No perturbation control (CN)	Treatment^a^	0.1969	0.6687	0.4517	0.0022	0.8001
	Control punctured (CP)	Treatment^a^	<0.0001	0.0027	0.3270	0.1776	0.1773
	Selected (S)	Treatment^a^	0.4262	0.0019	0.0001	0.0354	0.0012
	Treatments						
	AS	Lines^b^	0.6967	0.1663	0.6729	0.0462	
	H_2_O	Lines^b^	0.2074	0.2502	0.1727	0.0288	
	NON	Lines^b^	0.2717	0.8843	<0.0001	0.9560	
	A^c^	Lines^b^					0.0088
	B^c^	Lines^b^					0.8619
	C^c^	Lines^b^					0.3675
	D^c^	Lines^b^					0.4857
	E^c^	Lines^b^					0.0098

^a^Treatments: punctured with autoclaved spores (AS), punctured with sterile water (H_2_O), and no treatment (NON).

^b^Line types: selected (S), punctured with H_2_O each generation (CP), and no perturbation each generation (CN).

^c^A–E were *F*_1_ matings for the progeny development time assay (see Table 1).

**Table 3:** Average survival time of female and male flies, average total number of eggs, and average progeny production: lines and treatments.

Lines	Treatments	Mean (S.E.) female survival	Mean (S.E.) male survival	Mean (S.E.) egg number	Mean (S.E.) progeny number
No perturbation control (CN)	Autoclaved spores	31.35 (1.84)	40.50 (1.63)	577 (45.43)	185 (18.7)
No perturbation control (CN)	H_2_O	35.37 (0.84)	42.88 (0.64)	639 (47.48)	274 (17.6)
No perturbation control (CN)	NON	35.65 (0.84)	42.22 (1.64)	561 (37.49)	214 (18.1)
Control punctured (CP)	Autoclaved spores	27.92 (2.16)	40.10 (1.19)	636 (46.17)	225 (18.9)
Control punctured (CP)	H_2_O	37.17 (1.24)	47.65 (1.62)	699 (38.21)	262 (17.3)
Control punctured (CP)	NON	38.20 (1.24)	40.65 (1.83)	737 (50.09)	219 (17.5)
Selected (S)	Autoclaved spores	27.65 (1.68)	35.17 (2.83)	588 (55.27)	161 (17.5)
Selected (S)	H_2_O	29.37 (3.51)	43.44 (1.93)	763 (45.48)	210 (18.0)
Selected (S)	NON	30.64 (3.10)	41.57 (1.86)	874 (55.66)	222 (17.3)

Treatments: punctured with autoclaved spores (AS), punctured with sterile water (H_2_O), and no treatment (NON).

Line types: selected (S), punctured with H_2_O each generation (CP), and no perturbation each generation (CN).

**Table 4:** Average progeny emergence (eclosion) time from each of the following crosses applied to each line type.

Lines	Treatments	Mean (hours)	S.E.
No perturbation control (CN)	A	63.78	2.08
No perturbation control (CN)	B	63.17	2.78
No perturbation control (CN)	C	65.31	2.82
No perturbation control (CN)	D	64.14	2.58
No perturbation control (CN)	E	62.96	2.02
Control punctured (CP)	A	67.63	3.22
Control punctured (CP)	B	64.53	2.43
Control punctured (CP)	C	68.34	2.74
Control punctured (CP)	D	64.20	2.67
Control punctured (CP)	E	66.32	2.08
Selected (S)	A	71.60	3.61
Selected (S)	B	63.75	3.21
Selected (S)	C	68.45	3.50
Selected (S)	D	66.80	2.84
Selected (S)	E	70.68	3.08

Treatments: females punctured with autoclaved spores and mated with untreated males (A), males punctured with autoclaved spores and mated with untreated females (B), females punctured with sterile water and mated with untreated males (C), males punctured with sterile water and mated with untreated females (D), and untreated females mated with untreated males (E).

Line types: selected (S), punctured with H_2_O each generation (CP), no perturbation each generation (CN).
