# Supplementary material for: Drosophila melanogaster Selection for Survival of Bacillus cereus Infection: Life History Trait Indirect Responses
Source: Int J Evol Biol. 2012 Oct 9;2012:935970. doi: 10.1155/2012/935970 (PMC3474238; doi:10.1155/2012/935970)
Supplement: Supplementary file 1 — In the supplementary material, S 1a, S1b and S1c tables present the F values and degrees of freedom from the statistical analysis with using a mixed model ANOVA of female and male survival time (a), egg and progeny production (b) and progeny development time (c). Figures S2a and S2b present average percentage survival of the selected and control lines in which adult females (a) and males (b) were untreated. . [file 935970.f1.pdf]

S 1a. F values and degrees of freedom from the statistical analysis (mixed model ANOVA) of female and male survival time

Overall Effects	Lines/Treatments	Comparisons	Num DF of female survival	Den DF of female survival	F value of female survival	Num DF of male survival	Den DF of male survival	F value of male survival
Lines			2	6	0.93	2	6	0.70
Treatments			2	83	10.51	2	84	8.89
Lines*Treatments			4	83	2.00	4	84	1.69
	Lines							
	No Perturbation Control (CN)	Treatment [a]	2	83	1.66	2	84	0.40
	Control Punctured (CP)	Treatment [a]	2	83	12.58	2	84	6.34
	Selected (S)	Treatment [a]	2	83	0.86	2	84	6.73
	Treatments							
	AS	Lines [b]	2	83	0.36	2	84	1.83
	H_2O	Lines [b]	2	83	1.60	2	84	1.41
	NON	Lines [b]	2	83	1.32	2	84	0.12

a. Treatments: punctured with autoclaved spores (AS), punctured with sterile H_2O and no treatment (NON) ;

b. Line types: selected (S), punctured with H_2O each generation (CP), no perturbation each generation (CN)

S 1b. F values and degrees of freedom from the statistical analysis (mixed model ANOVA) of egg and progeny production

Overall Effects	Lines/Treatments	Comparisons	Num DF of egg production	Den DF of egg production	F value of egg production	Num DF of progeny production	Den DF of progeny production	F value of progeny production
Lines			2	6	7.79	2	6	3.55
Treatments			2	502	5.62	2	477	7.76
Lines*Treatments			4	502	2.74	4	477	1.68
	Lines							
	No Perturbation Control (CN)	Treatment [a]	2	502	0.80	2	477	6.21
	Control Punctured (CP)	Treatment [a]	2	502	1.12	2	477	1.73
	Selected (S)	Treatment [a]	2	502	9.14	2	477	3.36
	Treatments							
	AS	Lines [b]	2	502	0.40	2	477	3.09
	H_2O	Lines [b]	2	502	1.76	2	477	3.57
	NON	Lines [b]	2	502	11.63	2	477	0.04

a. Treatments: punctured with autoclaved spores (AS), punctured with sterile H_2O and no treatment (NON)；

b. Line types: selected (S), punctured with H_2O each generation (CP), no perturbation each generation (CN)

S 1c. F values and degrees of freedom from the statistical analysis (mixed model ANOVA) of progeny development time

Overall Effects	Lines/Treatments	Comparisons	Num DF of progeny development time	Den DF of progeny development time	F value of progeny development time
Lines			2	4	3.24
Treatments			4	234	3.81
Lines*Treatments			8	234	1.44
	Lines				
	No Perturbation Control (CN)	Treatment [a]	4	234	0.41
	Control Punctured (CP)	Treatment [a]	4	234	1.59
	Selected (S)	Treatment [a]	4	234	4.69
	Treatments				
	A [a]	Lines [b]	2	234	4.83
	B [a]	Lines [b]	2	234	0.15
	C [a]	Lines [b]	2	234	1.01
	D [a]	Lines [b]	2	234	0.72
	E [a]	Lines [b]	2	234	4.72

a. females punctured with autoclaved spores and mated with untreated males (A), males punctured with autoclaved spores and mated with untreated females (B), females punctured with sterile water and mated with untreated males (C), males punctured with sterile water and mated with untreated females (D), and untreated females mated with untreated males (E)
b. line types: selected (S), punctured with H_2O each generation (CP), no perturbation each generation (CN)

S2

a

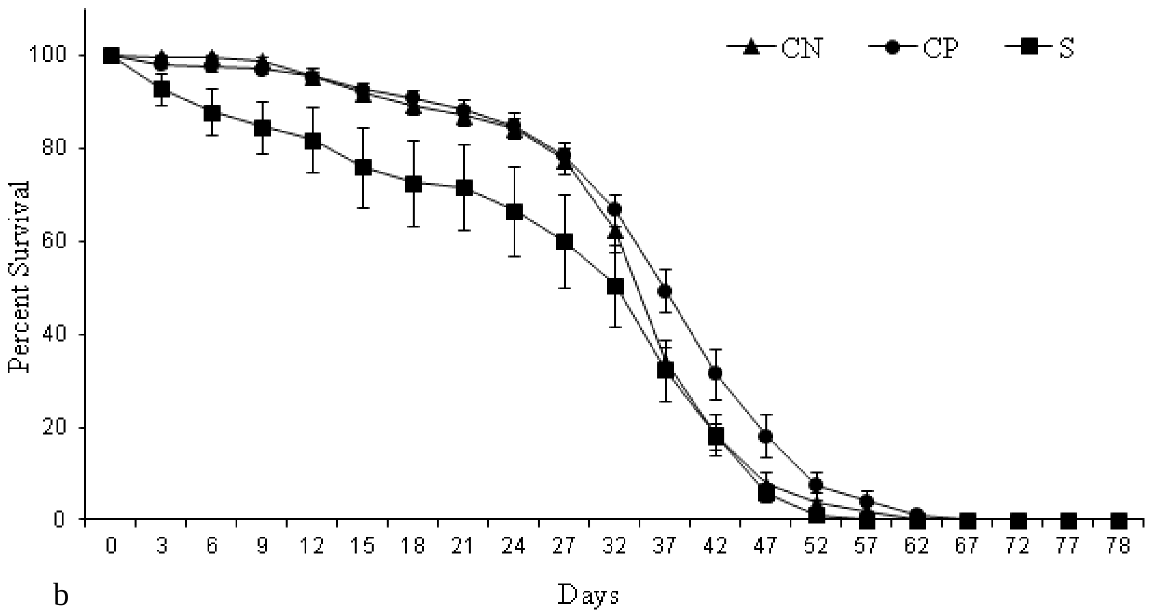

b

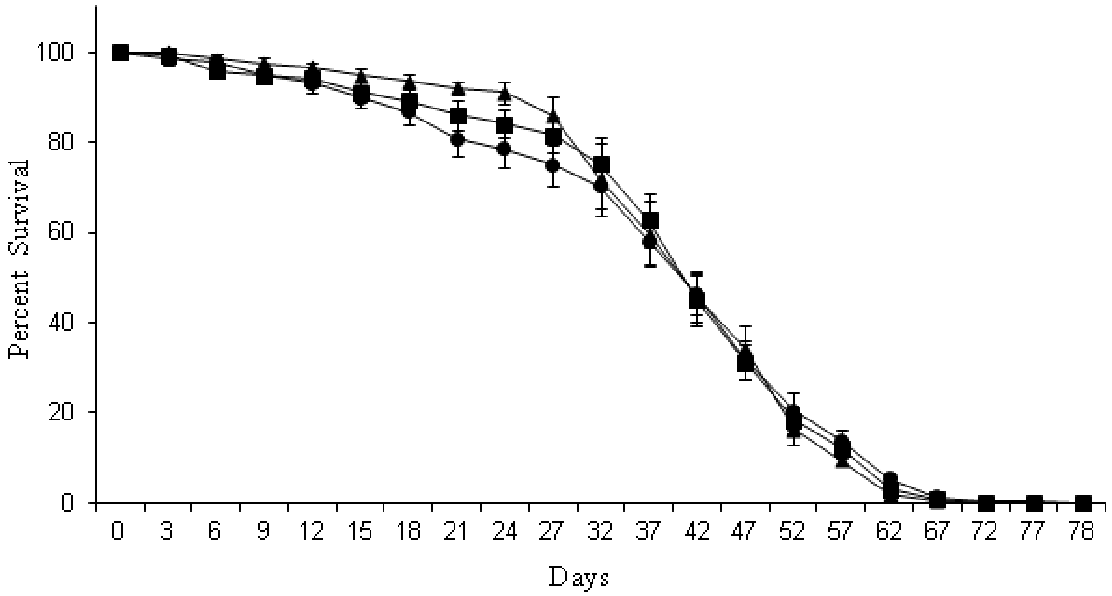

a: Average percentage survival of untreated adult females from the selected and control lines. The mean was determined from the replicate lines of the same type: S-selected lines, CP-lines punctured with H_2O (wound control), CN-no perturbation lines.

b: Average percentage survival of untreated adult males from the selected and control lines. The mean was determined from the replicate lines of the same type: S-selected lines, CP-lines punctured with H_2O (wound control), CN-no perturbation lines.
